# Polyphosphate Kinase Mediates Antibiotic Tolerance in Extraintestinal Pathogenic *Escherichia coli* PCN033

**DOI:** 10.3389/fmicb.2016.00724

**Published:** 2016-05-19

**Authors:** Jing Chen, Lijie Su, Xiangru Wang, Tao Zhang, Feng Liu, Huanchun Chen, Chen Tan

**Affiliations:** ^1^State Key Laboratory of Agricultural Microbiology, College of Veterinary Medicine, Huazhong Agricultural UniversityWuhan, China; ^2^School of Public Health, Guangzhou Medical UniversityGuangzhou, China; ^3^Key Laboratory for the Development of Veterinary Diagnostic Products, The Cooperative Innovation Center for Sustainable Pig Production, Ministry of Agriculture, Huazhong Agricultural UniversityWuhan, China

**Keywords:** ExPEC, antibiotic resistance, PPK, gene expression, RNA-seq, qPCR

## Abstract

Extraintestinal pathogenic *Escherichia coli* (ExPEC) causes a variety of acute infections in its hosts, and multidrug-resistant strains present significant challenges to public health and animal husbandry. Therefore, it is necessary to explore new drug targets to control *E. coli* epidemics. Previous studies have reported that *ppk* mutants of *Burkholderia pseudomallei* and *Mycobacterium tuberculosis* are more susceptible than the wild types (WTs) to stress. Therefore, we investigated the stress response to antibiotics mediated by polyphosphate kinase (PPK) in ExPEC strain PCN033. We observed that planktonic cells of a *ppk* knockout strain (Δ*ppk*) were more susceptible to antibiotics than was WT. However, biofilm-grown Δ*ppk* cells showed similar susceptibility to that of the WT and were more tolerant than the planktonic cells. During the planktonic lifestyle, the expression of genes involved in antibiotic tolerance (including resistance-conferring genes, and antibiotic influx, and efflux genes) did not change in the Δ*ppk* mutant without antibiotic treatment. However, the resistance-conferring gene *bla* and efflux genes were upregulated more in the WT than in the Δ*ppk* mutant by treatment with tazobactam. After treatment with gentamycin, the efflux genes and influx genes were upregulated and downregulated, respectively, more in the WT than in the Δ*ppk* mutant. The expression of genes involved in biofilm regulation also changed after treatment with tazobactam or gentamycin, and which is consistent with the results of the biofilm formation. Together, these observations indicate that PPK is important for the antibiotic stress response during the planktonic growth of ExPEC and might be a potential drug target in bacteria.

## Introduction

Extraintestinal pathogenic *Escherichia coli* (ExPEC) is a major cause of urinary tract infections in women, abdominal sepsis, and septicemia in elderly or immunocompromised individuals, and meningitis in newborns, with high morbidity and mortality (Gaschignard et al., 2011; Weston et al., 2011; Mellata, 2013). ExPEC strains commonly colonize domestic animals, such as pigs, chickens, and cattle, causing significant losses in animal husbandry and threating human health (Girardeau et al., 2003; Johnson et al., 2005; Bergeron et al., 2012). We previously investigated the prevalence of ExPEC in swine across China and detected ExPEC in 10.1% of porcine samples. The frequency of ExPEC isolated from pigs increased between 2004 and 2007 from 3.1 to 14.6% (Tan et al., 2012). The emergence of multidrug-resistant strains has significantly hindered the prevention and control of ExPEC epidemics (Sedláková et al., 2014; Sidrach-Cardona et al., 2014). Therefore, it is urgent that we identify new drug targets to control these increasing *E. coli* outbreaks.

Polyphosphate kinase (PPK) is an essential enzyme in polyphosphate (polyP) synthesis and has been implicated in many intracellular biological processes. *Pseudomonas aeruginosa* in which *ppk* was deleted showed impairments in motility, quorum sensing, and virulence (Rashid et al., 2000), compacted nucleoids, membrane distortion, extracellular polymer production, and a susceptibility to desiccation (Fraley et al., 2007). PPK also plays a prominent role in the stress response, and a *Burkholderia pseudomallei ppk* mutant was susceptible to hydrogen peroxide under oxidative stress conditions (Tunpiboonsak et al., 2010). A *ppk1* mutant strain of *Mycobacterium tuberculosis* displayed a survival defect in response to nitrosative stress, and the negligible levels of polyP were associated with its increased susceptibility to certain tuberculosis drugs (Singh et al., 2013). PPK is highly conserved in bacteria, but is absent in higher mammals (Brown and Kornberg, 2004), indicating that PPK has potential utility as an antibacterial drug target.

As an opportunistic pathogen, *E. coli* mainly causes acute infections in immunocompromised individuals (Chaudhuri and Henderson, 2012; Mellata, 2013); further, acute infections are associated with its planktonic growth mode (Li et al., 2014). Therefore, we explored the role of PPK in antibiotic resistance in the planktonic cells of ExPEC strain PCN033. Biofilm formation contributes to chronic bacterial infections, such as the recurrent pyelonephritis caused by uropathogenic *E. coli* in children (Tapiainen et al., 2014). Therefore, we also studied the role of PPK in antibiotic tolerance in biofilm-grown cells.

## Materials and methods

### Bacterial strains and culture conditions

The wild-type (WT) strain used in this study, PCN033, was isolated from a diseased swine in Hubei Province, Central China (Liu et al., 2015). The Δ*ppk* mutant was obtained by in-frame deletion with the suicide plasmid pRE112 (He et al., 2012). Details of both these strains and the primers used in this study are listed in Table S1 (available as Supplementary Data). The antibiotic susceptibility and biofilm formation assays were performed in MOPS broth (an inorganic phosphorus [P_i_]-limited medium) at 28°C (Neidhardt et al., 1974). The antibiotics used in the biofilm inhibition assay were added below the minimum inhibitory concentrations (MICs), and had no bactericidal effect on the planktonic cells.

### Growth characteristics

Fresh colonies of both strains were taken from Luria–Bertani (LB) agar plates, used to inoculate LB broth, and then cultured in MOPS in a shaker incubator for 12 h. The growth characteristics were monitored turbidimetrically at 600 nm on a spectrophotometer (Eppendorf, Hamburg, Germany) and the colony-forming units (cfu) were counted at 1 h intervals. The generation times were calculated with the formula (Penfold and Norris, 1912):

$$G=\frac{T}{Log_{2}^{\frac{b}{a}}}$$

where *G* is the generation time; *T* is the length of the logarithmic phase; *a* is the initial number of bacteria; and *b* is the final number of bacteria.

### Susceptibility assay

Each MIC was determined with a series of two-fold dilutions of the antibiotic in MOPS broth, according to the Clinical Laboratory and Standards Institute guidelines. A pre-grown inoculum of each strain was diluted in MOPS to a final concentration of 10^7^ cfu/mL, and the concentration of antibiotic added varied from 0.25 to 512 mg/L. The plates were incubated for 24 h, and the MICs were determined as the lowest antibiotic concentrations that produced no visible growth.

The susceptibility assay of the biofilms was performed as described previously (Benthall et al., 2015), with some modifications. The MIC on the biofilm was determined by allowing a biofilm to form in a 96-well-plate for 24 h. The unattached cells were washed off three times with 0.9% saline. The biofilm was incubated for 24 h with a range of antibiotic concentrations from 512 to 0.25 mg/L. The MIC was defined as the lowest antibiotic concentration at which no bacterial growth was detected. To determine the effect of the biofilm on the bacterial susceptibility to antibiotics, the viability of planktonic and biofilm-grown cells was calculated after the antibiotic treatments. About 10^7^ cfu were incubated with antibiotic concentrations of 2 × MIC for 3 h, and the cfu were then counted.

To quantify the bactericidal activity of these antibiotics on the biofilm, their activity percentage was assessed according to a previous report (Sánchez-Gómez et al., 2015), with some modifications. The ability of these antibiotics to remove the biofilm attached to the microplate was determined with crystal violet (CV) staining. For this purpose, the treated biofilm was stained with CV for 30 min at room temperature. The excess stain was then rinsed off with saline and the CV remaining on the biofilm was dissolved in 33% acetic acid. The absorbance was measured at 595 nm with a Synergy HT microplate reader (BioTek, USA). The activity percentage was calculated according to Sánchez-Gómez et al., with the formula:

$$Activity\;Percentage=\frac{\left(C-B)-(T-B\right)}{C-B}\text{ x 100}$$

where *C* is the absorbance of the control well-containing untreated biofilm; *T* is the absorbance of the well-containing treated biofilm; and *B* is the absorbance of the blank well (i.e., no biofilm).

### Biofilm formation assay

Static cultures of biofilm grown at 28°C were analyzed in flat-bottom 96-well-microtiter plates (Corning, USA) using CV. Briefly, approximately 10^7^ cfu were inoculated with sub-MIC antibiotics and cultured for 24 h. The unattached cells were then washed off as described above, fixed with absolute ethanol for 30 min, dried, and stained with 0.1% CV solution for 1 h. The microplates were then washed three times to remove any unattached CV. The CV in the stained biofilm was then dissolved in 33% acetic acid solution and the absorbance read at 595 nm. Each experiment was repeated twice with three technical replicates.

### RNA-seq assay and quantitative real-time (qRT)–PCR validation

RNA samples from each strain were prepared for RNA sequencing. Sequencing was performed on an Illumina Hiseq 2500 sequencer (Illumine Inc.) by Shanghai Hanyu Biotechnology Co., Ltd (Shanghai, China). The RNA-seq results were confirmed with qRT–PCR. Before qRT-PCR, the RNA of both strains was extracted with RNAiso Plus reagent (Takara, China). Any genomic DNA contamination was eliminated, and the RNA was reverse transcribed to cDNA with the PrimeScript™RT reagent Kit with gDNA Eraser (Takara). Quantitative real-time PCR was performed in triplicate in optical 96-well-reaction plates (Life Technologies, China) using Power SYBR Green PCR Master Mix (Life Technologies). The primers are listed in Table S1. The mRNA levels of the target genes were normalized to the internal 16S rRNA control with the ΔΔCt method (Kubista et al., 2006). The planktonic cells of the WT and Δ*ppk* mutant were cultured to an optical density at a wavelength of 600 nm (OD_600_) of about 0.5. Tazobactam or gentamycin was added at a concentration of 2 × MIC and the cells were incubated for 2 h. The cells were then collected for RNA extraction and qRT–PCR.

### Statistical analysis

Statistical analysis was performed with the SPSS software (SPSS, Inc., Chicago, IL, USA) on a Windows XP system. Biofilm formation, differential expression, and generation times were compared with one-way analysis of variance (ANOVA). Values are expressed as means ± *SD*, and statistically significant differences are marked with asterisks. To identify the levels of gene expression, the RNA-seq data were analyzed with an MA-plot-based method with the random sampling model (MARS) in the DEGseq software (http://www.bioconductor.org/packages/release/bioc/html/DESeq.html). Genes showing two-fold changes in expression, a false discovery rate < 0.001, and reads per kilobase per million >20 in at least one sample were considered to be differentially expressed under the conditions used.

## Results

### Growth characteristics

**Figure 1:**
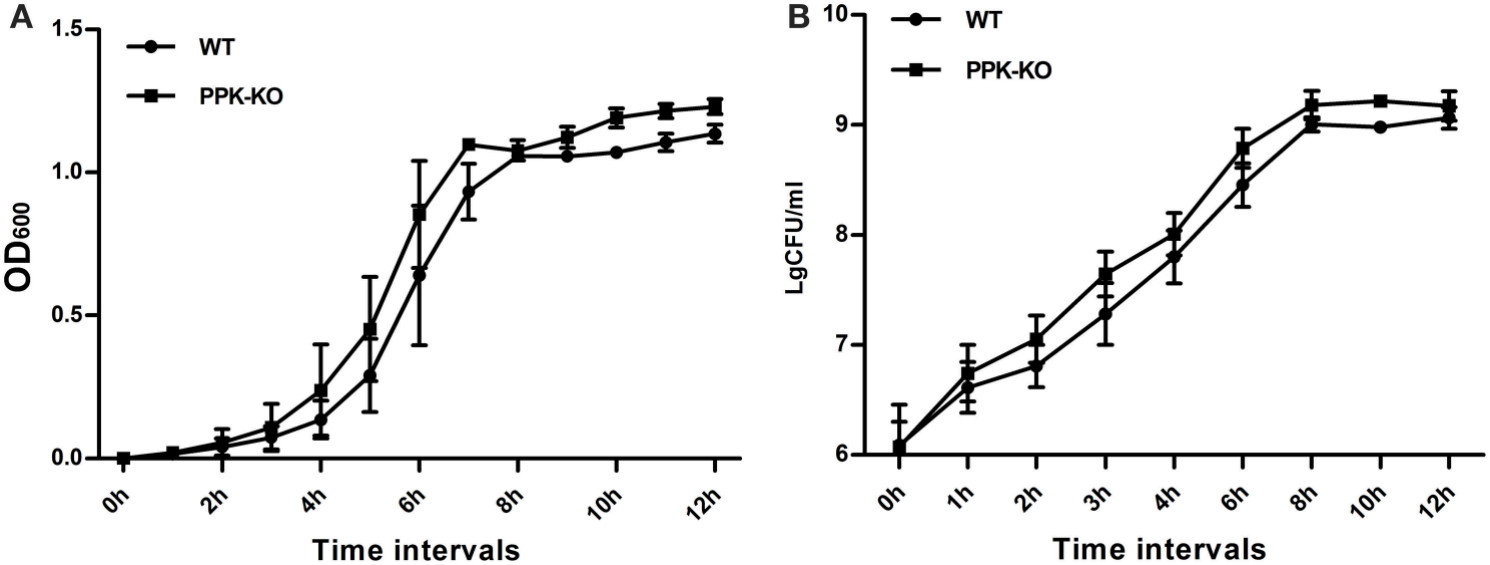
**Growth characteristics of *E. coli* strains PCN033 (wild type) and Δ*ppk* in MOPS broth: (A) optical density, and (B) colony-forming units**.

In MOPS minimal medium, the density of the Δ*ppk* cultures was slightly higher than that of the WT at each time point tested (Figure 1). However, the generation times, calculated from both the optical density and cfu, for Δ*ppk* (*G*_OD_ = 56.5 ± 4.73, *G*_CFU_ = 42.0 ± 1.88) were not significantly different (*P* > 0.05) from those for WT (*G*_OD_ = 56.3 ± 3.64, *G*_cfu_ = 45.9 ± 4.35).

### Planktonic Δ*ppk*cells are more susceptible to antibiotics than WT cells

As shown in Table 1, the 17 antibiotics screened in this study were categorized based on their targets: cell wall biosynthesis (type A), protein biosynthesis (type B), nucleotide metabolism (type C), and cell membrane (type D). There were no significant differences in the MIC values for the Δ*ppk* strain and WT in LB broth. However, in MOPS broth, Δ*ppk* was more susceptible to antibiotics than WT. Specifically, Δ*ppk* was much more susceptible to type B antibiotics than was WT, followed by type A antibiotics. Because the WT strain can accumulate more polyP in MOPS broth than in other medi (Ault-Riché et al., 1998), we performed all further assays in MOPS.

**Table 1:** **Assay of the susceptibility of planktonic cells to antibiotics (mg/L)**.

**Target (type)**	**antibiotic category**	**compound**	**MIC in LB**		**MIC change (fold)**	**MIC in MOPS**		**MIC fold (fold)**	**References**
			**PCN033**	**Δ*ppk***		**PCN033**	**Δ*ppk***		
Cell wall biosynthesis(A)	β-lactams	cefotaxime	2	1	2	16	4	4	Bush, 2012
		ceftazidime	256	256	1	512	512	1	
		ampicillin	>512	>512	1	>512	>512	1	
		cefazolin	>512	>512	1	>512	>512	1	
		tazobactam	>512	>512	1	>512	256	>2	
		ticarcillin	>512	>512	1	>512	>512	1	
	Glycopeptide	vancomycin	>512	>512	1	128	64	2	
Protein biosynthesis (B)	Aminoglycosides	gentamicin	>512	>512	1	256	32	8	Davis, 1987
		gentamicin sulfate	>512	>512	1	256	64	4	
		amikacin	>512	>512	1	>512	64	≥4	
	Macrolide	Erythromycin	512	512	1	512	128	4	Brisson-Noël et al., 1988
Nucleotide metabolism (C)	Quinolones	norfloxacin	>512	512	>1	512	512	1	Aldred et al., 2014
		levofloxacin	128	64	2	64	16	4	
	Sulfonamides	Trimethoprim	128	128	1	256	128	2	Pérez-Trallero and Iglesias, 2003
		sulfadiazine	>512	>512	>1	>512	>512	1	
	Nitrofurans	macrodantin	512	256	2	128	128	1	Hof, 1988
Membrane (D)	Lipopeptde	polymyxin B	0.5	≤ 0.25	≥2	2	1	2	Grau-Campistany et al., 2015

### Biofilm-grown Δ*ppk* and WT cells are similarly tolerant and more tolerant than planktonic cells

**Figure 2:**
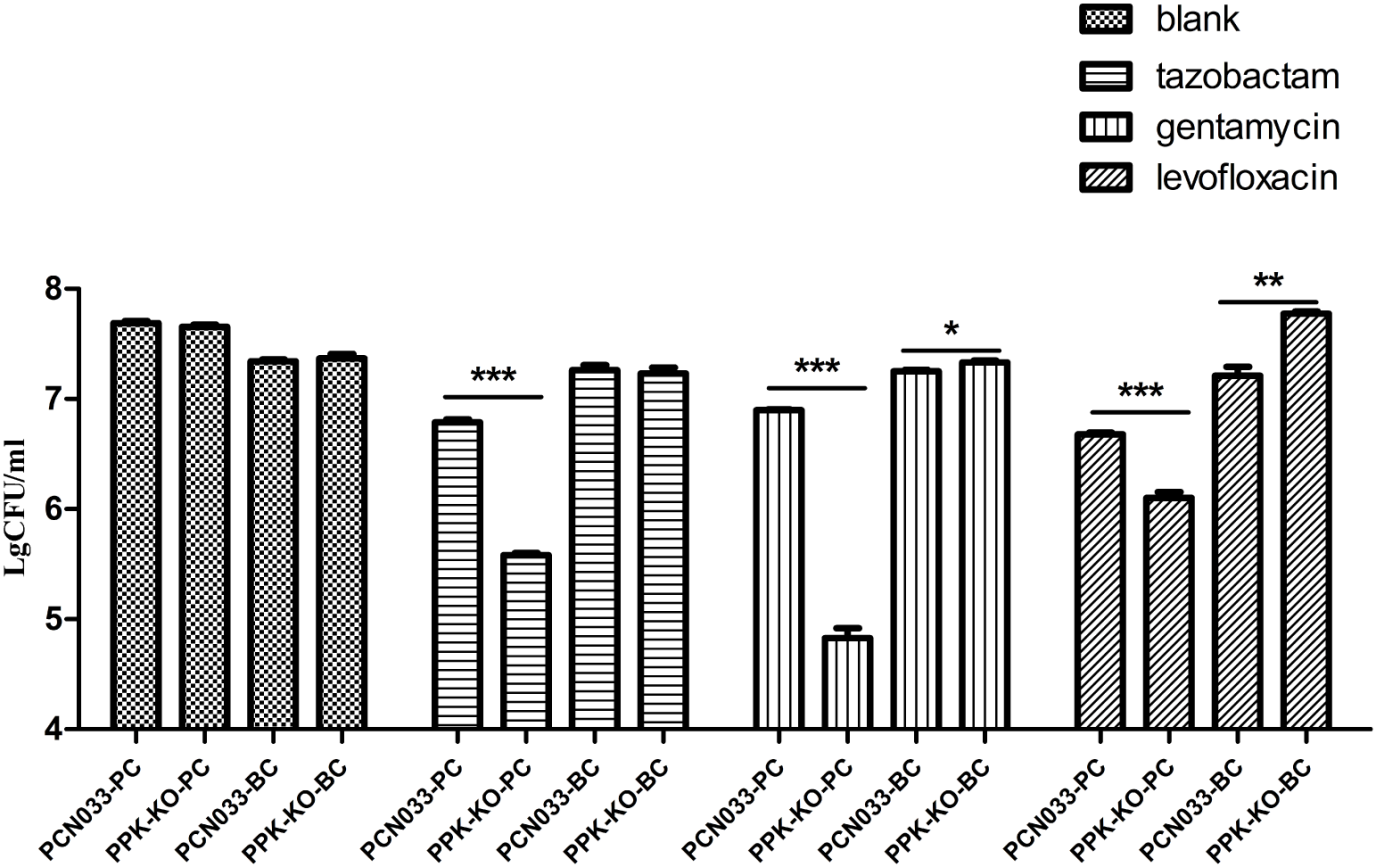
**Antibiotic killing assay of planktonic and biofilm-grown cells**. PCN033-PC indicates planktonic WT cells; PPK-KO-PC indicates planktonic cells of the Δ*ppk* mutant; PCN033-BC indicates biofilm-grown WT cells; PPK-KO-BC indicates biofilm-grown cells of the Δ*ppk* mutant; blank indicates no antibiotic treatment. ^***^p < 0.000, ^**^p < 0.01, ^*^p < 0.05.

To clarify the role of biofilms in antibiotic resistance, an MIC assay using biofilm-grown cells was performed as described previously (Benthall et al., 2015). As shown in Table 2, biofilm-grown Δ*ppk* cells showed almost no difference from WT cells in their antibiotic susceptibility, and biofilm-grown cells of both strains were more tolerant than the corresponding planktonic cells. Consistent with this, there was no significant difference in the ability of antimicrobial compounds to kill biofilm-grown cells of the WT and Δ*ppk* strains (Figure S1). However, more planktonic cells of the Δ*ppk* strain were killed than WT cells (Figure 2). In a future study, we will investigate the role of PPK in antibiotic tolerance within the planktonic growth mode.

**Table 2:** **Assay of the susceptibility of biofilm-grown cells to antibiotics (mg/L)**.

**Target (type)**	**antibiotic category**	**compound**	**MIC for biofilm**		**MIC change (fold)**
			**PCN033**	**Δ*ppk***	
Cell wall biosynthesis (A)	β-lactams	cefotaxime	128	256	0.5
		ceftazidime	512	512	1
		ampicillin	>512	>512	1
		cefazolin	>512	>512	1
		tazobactam	512	>512	≤ 0.5
		ticarcillin	>512	>512	1
Protein biosynthesis (B)	Aminoglycosides	gentamicin	512	512	1
		gentamicin sulfate	512	256	2
		amikacin	512	512	1
	Macrolide	erythromycin	512	512	1
	Quinolones	norfloxacin	512	512	1
		levofloxacin	32	32	1
Nucleotide metabolism (C)	Sulfonamides	Trimethoprim	512	512	1
		sulfadiazine	>512	>512	1
	Nitrofurans	macrodantin	512	512	1
Membrane (D)	Lipopeptde	polymyxin B	4	4	1

### Expression of antibiotic-resistance genes without antibiotic treatment

**Figure 3:**
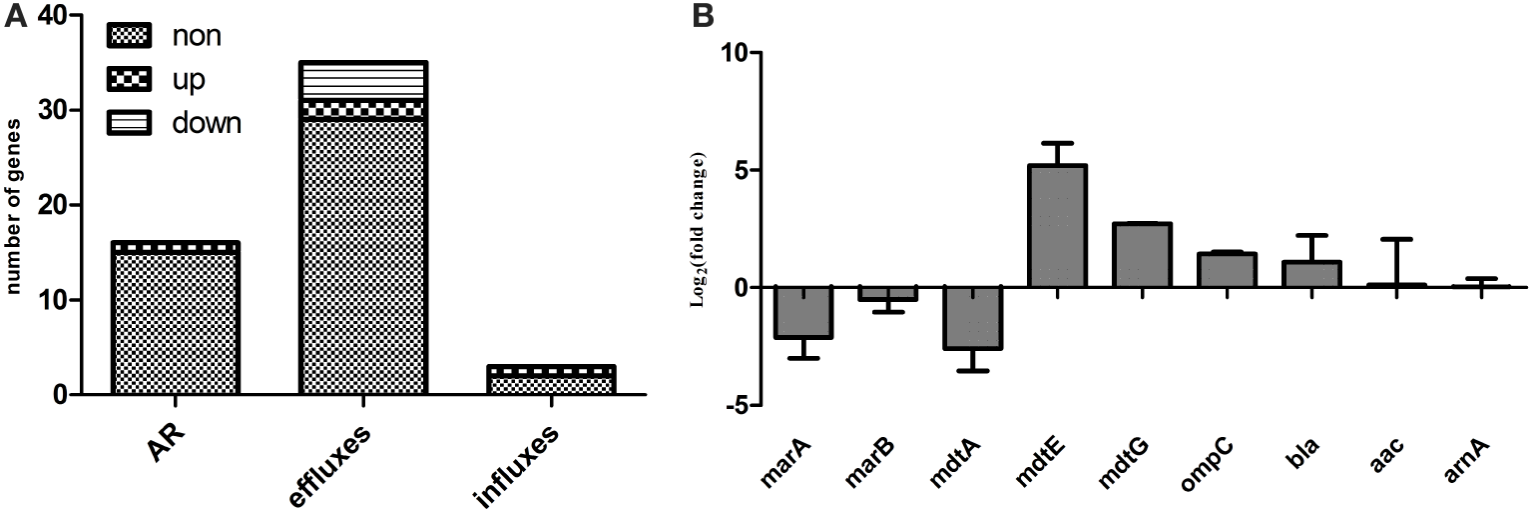
**Expression levels of genes involved in antibiotic resistance, antibiotic efflux, or antibiotic influx. (A)** Expression levels determined with RNA-seq; **(B)** qRT–PCR confirmation. AR indicates genes that confer resistance, including beta-lactamase *bla*, aminoglycoside 3′N-acetyltransferase III (*aac*) etc.; efflux genes include *acrAB–tolC, acrDEF, cusCFBA, emrAB, emrKY, mdtABCEF*, etc.; and influx genes include *ompC, ompF*, and *phoE*, details are available in Table S2.

RNA-seq data for the Δ*ppk* and WT strains regarding the expression of genes involved in antibiotic resistance, including resistance-conferring genes and antibiotic efflux and influx genes, are presented in Table S2. Of 53 genes known to be involved in antibiotic resistance or multidrug resistance, the expression of one resistance-conferring gene (*tetB*), two efflux genes (*mdtE* and *mdtG*), and one influx gene (*ompC*) was upregulated, and the expression of three efflux genes (*marA, marB*, and *mdtA*) was downregulated in the mutant compared with their expression in WT (Figure 3A). The expression of some of these genes was confirmed with qRT–PCR (Figure 3B).

### Expression of resistance-conferring genes after antibiotic treatment

**Figure 4:**
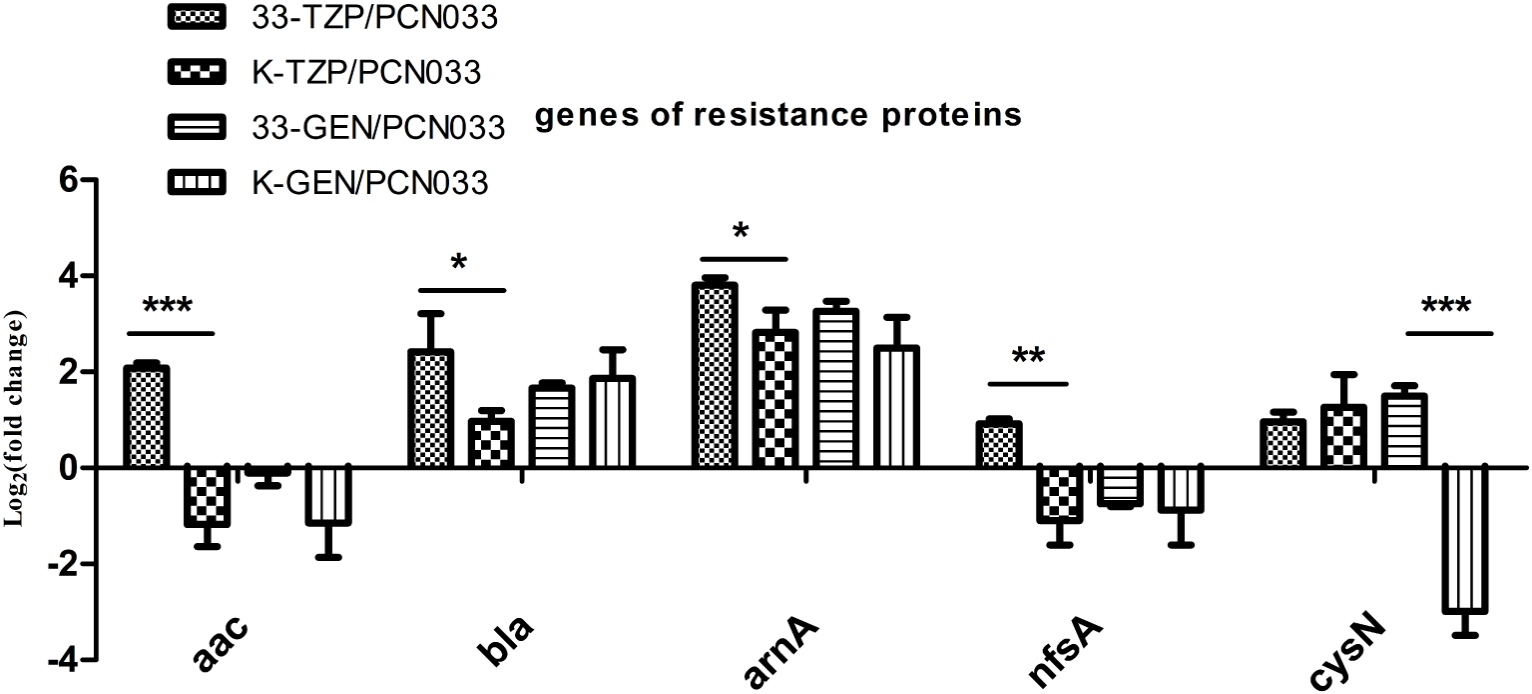
**Expression levels of genes that confer resistance, during tazobactam and gentamycin treatment**. Expression levels were compared with those of WT (PCN033). 33-TZP/PCN033 indicates the expression levels in PCN033 during tazobactam treatment compared with those in untreated PCN033; K-TZP/PCN033 indicates expression levels in the Δ*ppk* mutant during tazobactam treatment compared with those in untreated PCN033; 33-GEN/PCN033 indicates expression levels in PCN033 during gentamycin treatment compared with those in untreated PCN033; K-GEN/PCN033 indicates expression levels in the Δ*ppk* mutant during gentamycin treatment compared with those in untreated PCN033. ^***^p < 0.000, ^**^p < 0.01, ^*^p < 0.05.

Five resistance-conferring genes were selected for analysis when WT was treated with tazobactam, as shown in Figure 4. The expression of beta-lactamase (*bla*) was upregulated, as was that of aminoglycoside 3′N-acetyltransferase III (*aac*), fused UDP-L-Ara4N formyltransferase (*arnA*), and nitroreductase A (*nfsA*). The *bla, arnA*, and sulfate adenylyltransferase (*cysN*) genes were also upregulated in the Δ*ppk* mutant after tazobactam treatment, but their expression was higher in WT than in the Δ*ppk* mutant. The expression of resistance-conferring gene was not significantly altered by gentamycin treatment, except for *cysN*.

### Expression of genes involved in antibiotic transport was altered more strongly in WT than in the Δ*ppk* mutant

**Figure 5:**
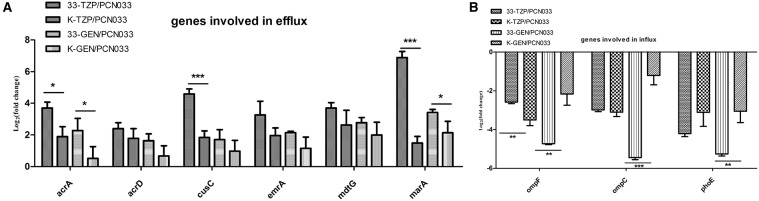
**Expression levels of genes involved in antibiotic efflux and influx during tazobactam and gentamycin treatments. (A)** Expression of efflux genes, **(B)** expression of influx genes. Expression levels of the genes were compared with those of WT (PCN033). 33-TZP/PCN033 indicates the expression levels in PCN033 during tazobactam treatment compared with those in untreated PCN033; K-TZP/PCN033 indicates the expression levels in the Δ*ppk* mutant during untreated PCN033.K-GEN/PCN033 indicates the expression levels in the Δ*ppk* mutant during gentamycin treatment compared with those in untreated PCN033.^***^p < 0.000, ^**^p < 0.01, ^*^p < 0.05.

The expression of six genes involved in antibiotic efflux was determined, as shown in Figure 5A. The efflux genes were upregulated in both the WT and Δ*ppk* mutant strains by tazobactam and gentamycin. However, the expression of *acrA, cusC*, and *marA* was higher in WT than in the Δ*ppk* mutant after treatment with tazobactam. After treatment with gentamycin, the expression of *acrA* and *marA* was also higher in WT than in the Δ*ppk* mutant. The expression of three genes involved in antibiotic influx was determined, as shown in Figure 5B. All the genes tested were downregulated by antibiotic treatment. However, *ompF* expression decreased more strongly in WT than in the Δ*ppk* mutant when treated with tazobactam. The expression of *ompF, ompC*, and *phoE* also decreased more strongly in WT than in the Δ*ppk* mutant after treatment with gentamycin.

### Effects of tazobactam and gentamycin in biofilm formation

**Figure 6:**
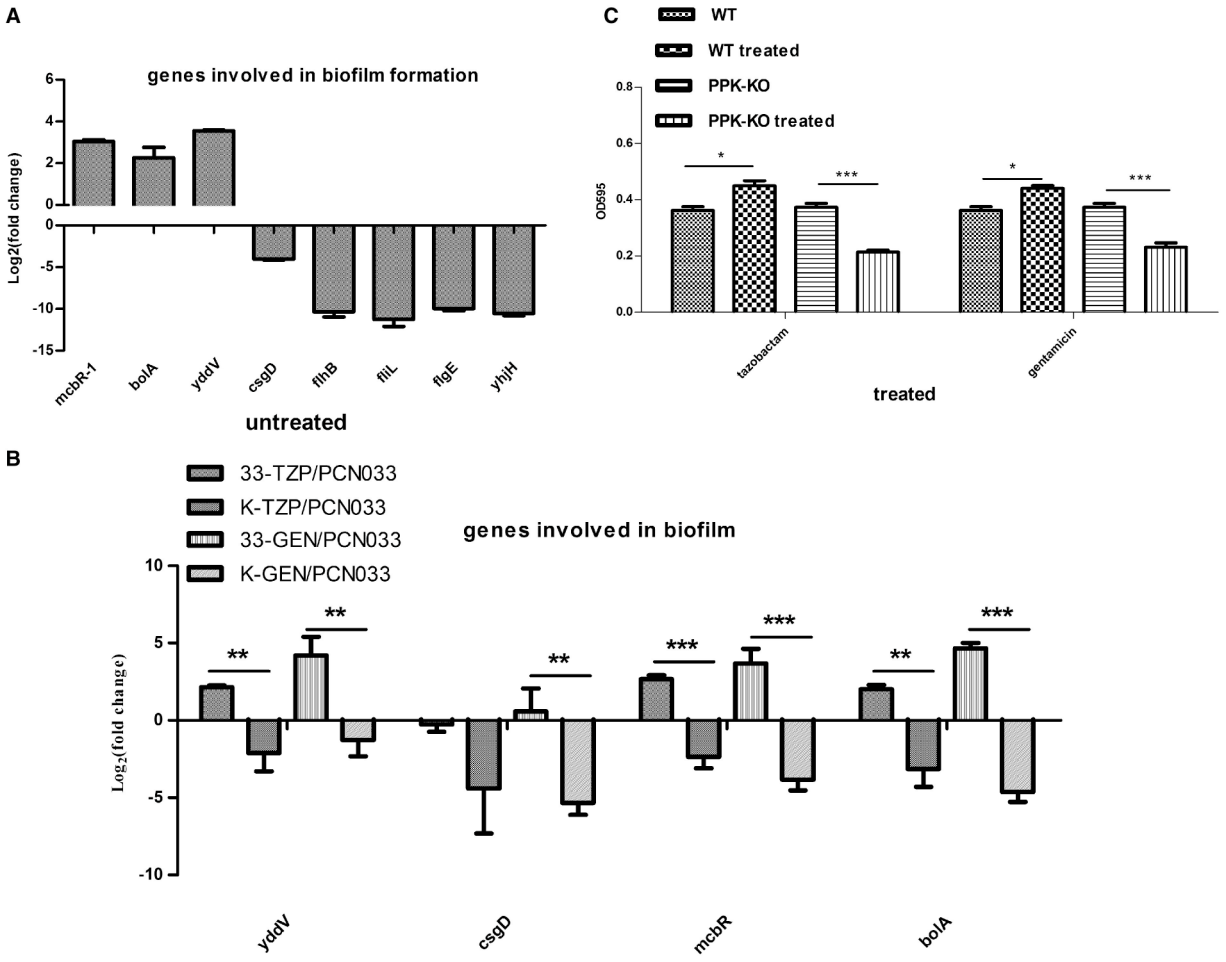
**Biofilm formation assay. (A)** Validation of the expression of genes involved in biofilm formation. **(B)** Expression levels of genes involved in biofilm regulation during tazobactam and gentamycin treatment. **(C)** Biofilm formation during tazobactam or gentamycin treatment. Expression levels of genes were compared with those in WT (PCN033). 33-TZP/PCN033 indicates the expression levels in PCN033 during tazobactam treatment compared with those in untreated PCN033; K-TZP/PCN033 indicates the expression levels in the Δ*ppk* mutant during tazobactam treatment compared with those in untreated PCN033; 33-GEN/PCN033 indicates the expression levels in PCN033 during gentamycin treatment compared with those in untreated PCN033; K-GEN/PCN033 indicates the expression levels in the Δ*ppk* mutant during gentamycin treatment compared with those in untreated PCN033. WT indicates PCN033 without antibiotic treatment; WT treated indicates PCN033 with antibiotic treatment; PPK-KO indicates the Δ*ppk* mutant without antibiotic treatment; PPK-KO treated indicates the Δ*ppk* mutant with antibiotic treatment. ^***^p < 0.000, ^**^p < 0.01, ^*^p < 0.05.

The RNA-seq data showed that the transcription levels of some genes associated with biofilm formation were altered in the Δ*ppk* strain (Tables S3, S4), especially those encoding the flagella cluster, which simultaneously promotes biofilm generation and impedes biofilm maturation (Laverty et al., 2014). The expression of fimbrial and curli genes was also reduced in the Δ*ppk* mutant. The expression levels of some genes were confirmed with qRT–PCR (Figure 6A). The expression of four genes (*yddV, mcbR, bolA*, and *csgD*) involved in biofilm regulation was determined during antibiotic treatment, as shown in Figure 6B (Laverty et al., 2014; Lord et al., 2014; Dressaire et al., 2015; Wu et al., 2015). The expression of *yddV, mcbR*, and *bolA* was upregulated in WT but downregulated in the Δ*ppk* mutant when treated with tazobactam, and all four genes were upregulated in WT but downregulated in the Δ*ppk* mutant when treated with gentamycin. Biofilm formation was also evaluated in the presence of tazobactam or gentamycin, as shown Figure 6C. Biofilm formation increased in WT planktonic cells but decreased in Δ*ppk* planktonic cells when treated with antibiotics.

## Discussion

Multidrug-resistant strains of ExPEC present significant challenges to public health and animal husbandry (Girardeau et al., 2003; Johnson et al., 2005; Bergeron et al., 2012). Because pathogenic *E. coli* mainly causes acute infections in its planktonic growth mode (Li et al., 2014), we initially investigated the role of PPK in the antibiotic resistance of ExPEC in the planktonic growth mode. We investigated in detail its susceptibility to different types of antibiotics, mediated by PPK, in *E. coli*. We found that PPK is very important in aminoglycoside tolerance, regulating the expression levels of antibiotic efflux and influx genes in the planktonic growth mode. Our findings indicate that PPK could have utility as a novel antimicrobial drug target.

As reported previously, resistance-conferring proteins and antibiotic efflux and influx porins play important roles in multidrug-resistance. Efflux proteins contribute to antibiotic tolerance by transporting compounds to the extracellular environment, whereas influx proteins have the opposite effect (Wilson, 2014). The expression levels of these genes were determined with RNA-seq, and showed that without antibiotic treatment, they did not differ significantly between WT and the Δ*ppk* mutant. Because PPK is reported to play prominent roles in the stress responses elicited by other stimuli (Alcántara et al., 2014), we investigated the role of PPK in the antibiotic stress response. Gentamycin and tazobactam were selected to treatthe planktonic cells of WT and the Δ*ppk* mutant. With gentamycin treatment, the expression of the efflux genes *acrA* and *marA* increased more strongly in WT than that in the Δ*ppk* mutant, and the influx porin genes *ompC* and *ompF* decreased more strongly in WT than in the Δ*ppk* mutant. Gentamycin binds the 30S ribosomal subunit and interrupts protein synthesis, thus inhibiting bacterial multiplication (Wargo and Edwards, 2014). According to Gray et al., compounds that interrupt protein metabolism cause intracellular polyP accumulation (Gray et al., 2014). Because it is a high-energy phosphate compound, polyP can be used to phosphorylate the response regulators of two-component systems to regulate gene expression (Sureka et al., 2007). As reported previously, the two-component systems CpxR and BaeR are implicated in antibiotic resistance by regulating the efflux genes of the *acr* operon and *mar* operon (Hu et al., 2011; Weatherspoon-Griffin et al., 2014; Pletzer et al., 2015). We speculated that phosphorylation of BaeR or CpxR using polyP as phosphate donar to modulate expression of *acrA* and *marA* during gentamycin treatment. The expression of porin genes *ompF* and *ompC* is upregulated by cAMP (Dalhoff, 1983), and the level of cAMP is negatively regulated by polyP, which potently inhibits the activity of the class III adenylate cyclases (Guo et al., 2009). Therefore, we speculated that the expression of *ompC* and *ompF* was downregulated by polyP during gentamycin treatment. Therefore, polyP may influence gentamycin tolerance by regulating the expression of antibiotic efflux and influx genes.

With tazobactam treatment, the expression of the resistance gene *bla* and efflux genes *acrA, cusC*, and *marA* was upregulated. Tazobactam binds to the periplasmic β-lactamase, and the efflux pump is implicated in resistance to beta-lactams and beta-lactamase inhibitors (Zhanel et al., 2014). However, there are insufficient data to clarify the role of PPK in regulating the expression of efflux pump genes induced by β-lactams. It will be interesting to explore the role of PPK in this process.

Biofilms contribute to antibiotic tolerance and chronic infection; thus, we also investigated the role of PPK in the antibiotic resistance of biofilm-grown cells. We observed that biofilm formation was impaired in the Δ*ppk* mutant when treated with antibiotics. The genes involved in biofilm formation (such as those encoding the fimbriae cluster, flagella cluster, and biofilm regulators BolA and McbR), were downregulated in the Δ*ppk* mutant by both antibiotic treatments. PolyP acts as a “chemical chaperone”, stabilizing cytoplasmic proteins intracellularly, similarly to heat shock proteins (Gray et al., 2014), and chaperones are known to be involved in biofilm formation. For example, the chaperone CsgE directs the intracellular localization of CsgA, the major subunit of the extracellular amyloid protein known as curli, which is essential for biofilm formation (Andersson et al., 2013). The FliS protein acts as a chaperone for FliC, a flagellar structural protein that promotes biofilm generation (Xu et al., 2014). The universal heat shock protein chaperones are also implicated in biofilm formation by fungi, such as *Candida albicans* (Robbins et al., 2011; Becherelli et al., 2013), and by Gram-negative bacteria, such as *E. coli* (Grudniak et al., 2013). Ultimately, these different effects reduce biofilm impairment during antibiotic treatment. Interestingly, PPK did not affect the antibiotic tolerance of ExPEC in the biofilm growth mode. Biofilms manifest antibiotic tolerance through many different mechanisms, including preventing the passage of antimicrobial compounds into the cytoplasm and possessing densely adherent growth (Qu et al., 2010). As reported previously, the planktonic and biofilm modes of growth are two distinct bacterial “lifestyles” (Chua et al., 2014), so it will be interesting to explore the roles of PPK in these distinct contexts.

## Author contributions

JC and CT designed and supervised the research project. JC wrote the paper, and LS, XW, HC, and TZ revised the manuscript. JC, TZ, and FL performed the experiments. JC and LS processed the data and performed the statistical analysis. All the authors have read and approved the manuscript.

## Funding

This work was supported by grants from the National Natural Science Foundation of China (grant numbers 31472201, 31030065, and 31421064) and the Programme for Introducing Talents of Discipline to Universities (grant number B12005).

### Conflict of interest statement

The authors declare that the research was conducted in the absence of any commercial or financial relationships that could be construed as a potential conflict of interest.

## Supplementary material

Figure S1 **Percentage activity of different antibiotics in removing biofilm. (A)** Targeting the cell wall, **(B)** targeting protein biosynthesis, and **(C)** targeting nucleotide metabolism.

Table S1 **Bacteria strains and primers**.

Table S2 **Transcription level of genes involved in antibiotic and multi-drug resistance**.

Table S3 **Expression of genes implicated in biofilm**.

Table S4 **Genes involved in biofilm formation (part results of RNA-seq)**.
