# Supplementary figures and images for: Polyphosphate Kinase Mediates Antibiotic Tolerance in Extraintestinal Pathogenic Escherichia coli PCN033
Source: Front Microbiol. 2016 May 19;7:724. doi: 10.3389/fmicb.2016.00724 (PMC4871857; doi:10.3389/fmicb.2016.00724)

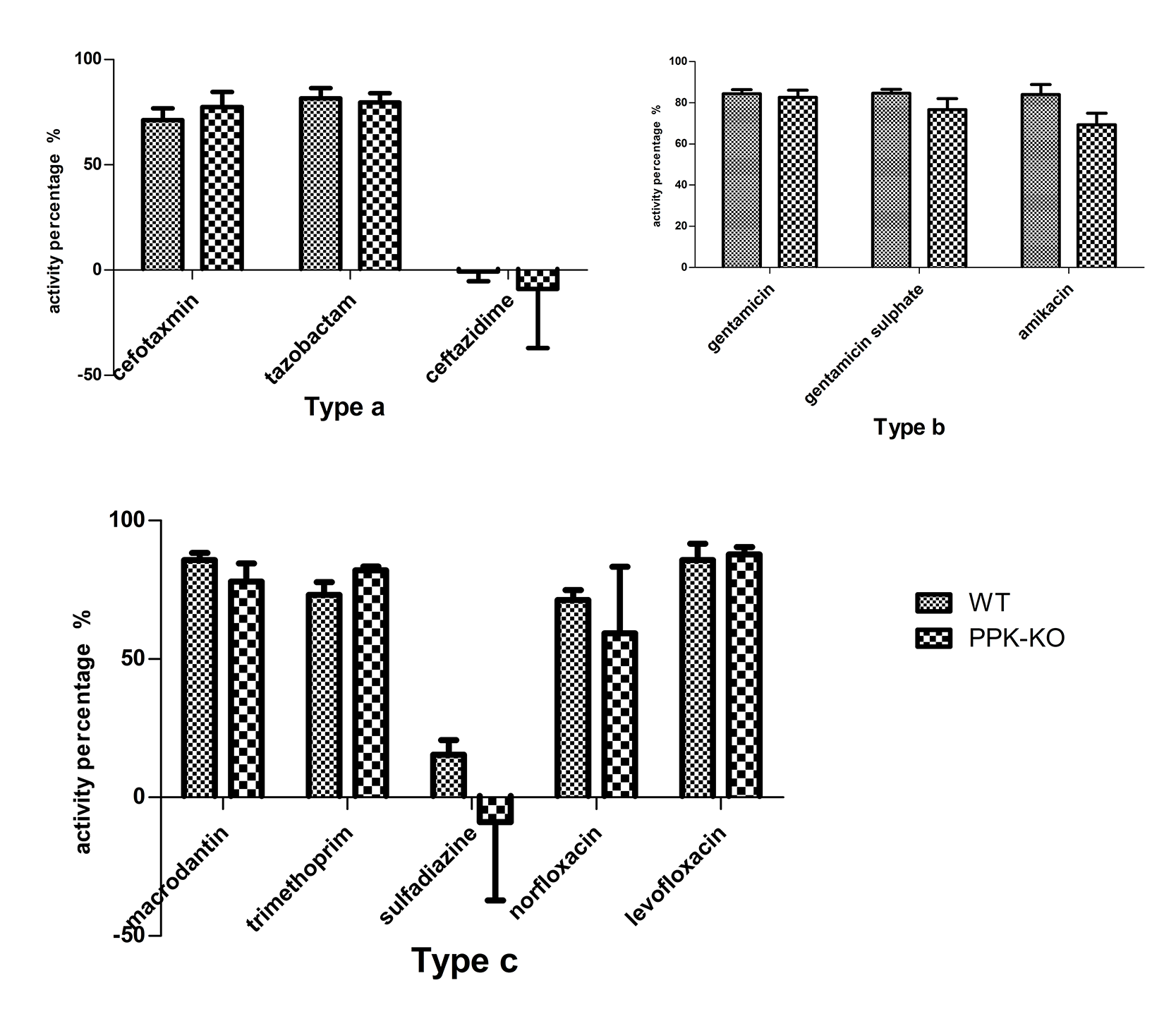

Supplement: Figure S1 — Percentage activity of different antibiotics in removing biofilm. (A) Targeting the cell wall, (B) targeting protein biosynthesis, and (C) targeting nucleotide metabolism. [file Image1.tif]
