# Supplementary material for: Polyphosphate Kinase Mediates Antibiotic Tolerance in Extraintestinal Pathogenic Escherichia coli PCN033
Source: Front Microbiol. 2016 May 19;7:724. doi: 10.3389/fmicb.2016.00724 (PMC4871857; doi:10.3389/fmicb.2016.00724)
Supplement: Table S1 — Bacteria strains and primers. [file Table1.DOCX]

Table S1. bacteria strains and primers

| strains, plasmids and primers | Description | source and reference |
| --- | --- | --- |
| PCN033 |  | isolated from diseased swine |
| X7213 | Thi-1 thr-1 leuB6 fhuA21 lacY1 glnV44 △asdA4 recA1 RP4 2-Tc∷Mu[λpir] Kmr | Yang He et al., 2012 |
| PPK-KO | PCN033 PPK-KO (+86 bp to + 1965 bp from the ATG of *ppk* ) | this study |
| pRE112 | Suicide vector, *pir*-dependent *ori* R6K, Cmr, SacB | Yang He et al., 2012 |
| ppk-P1 | 5’ *GG***GGTACC** ACCGACCCGCATTTACGT 3’ | this study |
| ppk-P2 | 5’ CGTAGCGATTACTGAGTTCTTTATTTGTCCGCCGCTTCCT 3’ | this study |
| ppk-P3 | 5’ AGGAAGCGGCGGACAAATAAAGAACTCAGTAATCGCTACG | this study |
| ppk-P4 | 5’ *C***GAGCTC**GATATTCAACCCGACCTCAT 3’ | this study |
| ppk-orf-1 | 5' *GGAATTC***CATATG**ATGGGTCAGGAAAAGCTAT 3' | This study |
| ppk-orf-2 | 5' *CG***GGATCC**CGTGTATTGGCATAGGGT 3' | This study |
| flhB-1 | 5' ATGGATCTGGTAGGGCTATG 3' | this study |
| flhB-2 | 5' AGGGTCGCCTTCGCTTTG 3' | this study |
| fliI-1 | 5' CGTCTGACTCGCTGGCTAA 3' | this study |
| fliI-2 | 5' CTTTCTACTTCGTGCGTTTCG 3' | this study |
| flgE-1 | 5' TTCTCAAGCGGTTAGCG 3' | this study |
| flgE-2 | 5' GGTCGTGCCATCGGTAAAG 3' | this study |
| YhjH-1 | 5' CGGATGGAGGTTGTGAAAGA 3' | this study |
| YhjH-2 | 5' ACGCAGCCAGGGAAGACG 3' | this study |
| yddV-1 | 5' CAATCAAACCATGCGTAATAC 3' | this study |
| yddV-2 | 5' GGTTAAGTAATTTCGTCAG 3' | this study |
| bolA-1 | 5' GTTCCAACCCGTATTCCT 3' | this study |
| bolA-2 | 5' CGCATGAACGGTAGTAGAGA 3' | this study |
| mcbR-1 | 5' TTGAAAACCTCACCCCGC 3' | this study |
| mcbR-2 | 5' GACCCACAGTTGCTCAATCAT 3' | this study |
| csgD-1 | 5' TCAGGGCGATCTTGTTTTAC 3' | this study |
| csgD-2 | 5' TCAACCCGCACAGTCACTAC 3' | this study |
| aac-1 | 5' GATAATGTCGGGCAATCAGGT 3' | this study |
| aac-2 | 5' CTTGATGGTCGGAAGAGGC 3' | this study |
| bla-1 | 5' GCTCCACATTTGCTGCC 3' | this study |
| bla-2 | 5' CATAGCCCCAGGTAAAGTAAT 3' | this study |
| arnA-1 | 5' GGAGCGTGATTTCTGTTG 3' | this study |
| arnA-2 | 5' CAGGCAGGCTGGCTATTC 3' | this study |
| cysN-1 | 5' GAAGCCTGGATGATTGCG 3' | this study |
| cysN-2 | 5' TGCCGTGACGTTTACTGTC 3' | this study |
| nfsA-1 | 5' CGTTTACCGGCCTCCATT 3' | this study |
| nfsA-2 | 5' GGCGGATATGATCGCTCT 3' | this study |
| mdtE-1 | 5' CACCCAGTTGAATGAAGCA 3' | this study |
| mdtE-2 | 5' CACCGACGATTTCCCGCT 3' | this study |
| mdtA-1 | 5' GATGATGCGCTGTTTCCC 3' | this study |
| mdtA-2 | 5' TGCTGACCTTGTTTTCGCT 3' | this study |
| marA-1 | 5' AAAGCTGAAGGAAAGTAACGAG 3' | this study |
| marA-2 | 5' GTATTTATGCGGCGGAAC 3' | this study |
| marB-1 | 5' AACCACTTTTATCCGCAATAG 3' | this study |
| marB-2 | 5' CACGACATTGGCACAGGAAG 3' | this study |
| LDH-1 | 5' TGTTTGAAATAATCCACCAG 3' | this study |
| LDH-2 | 5' GGTCACCTGTTCCCCAGCCAT 3' | this study |
| acrA-F | 5' TCGCCTGCTTTCAGACCT3' | this study |
| acrA-R | 5' CCAAACGCTATTTTAGTCCC3' | this study |
| acrD-f | 5' TATGGAATCGTTAGTGAAGCA3' | this study |
| acrD-r | 5' CCAGCAAGGAAATAGCGTA3' | this study |
| cusC-f | 5' CTGGAGCGGCAATCTTAA3' | this study |
| cusC-r | 5' GGCTTCCTCAGTGGCTAAAT3' | this study |
| emrA-f | 5' CGTCTGCTCCTGCTTCTC3' | this study |
| emrA-r | 5' TTTACAAAATCGGTGTTATCG3' | this study |
| ompF-f | 5' TGTAAGCGATGGACGGAC3' | this study |
| ompF-r | 5' TCTACCTGGCAGCGAACTA3' | this study |
| ompC-f | 5' CGATACCAAAGCCTTCATAA3' | this study |
| ompC-r | 5' GACCTACCGTAACACCGACT3' | this study |
| phoE-f | 5' GCCATCTTTACTGTCGTTATCA3' | this study |
| phoE-r | 5' ATGGGCATTGTGGCATCT3' | this study |

the bold capitals are restriction sites and the italic capitals are protective bases, while capitals with underlines are fusing primers for overlapping PCR to fuse the upstream arm and the downstream arm of the gene ppk.
