# Supplementary material for: Polyphosphate Kinase Mediates Antibiotic Tolerance in Extraintestinal Pathogenic Escherichia coli PCN033
Source: Front Microbiol. 2016 May 19;7:724. doi: 10.3389/fmicb.2016.00724 (PMC4871857; doi:10.3389/fmicb.2016.00724)
Supplement: Table S2 — Transcription level of genes involved in antibiotic and multi-drug resistance. [file Table2.DOCX]

Table S2. Transcription level of genes involved in antibiotic and multi-drug resistance

| Aminoglycoside 3'-phosphotransferase, aph | 0.1772455 |  | aminoglycoside | Glenn et al., 2014; |
| --- | --- | --- | --- | --- |
| Aminoglycoside 6''-phosphotransferase, strB | 0.3171906 |  | aminoglycoside |  |
| Aminoglycoside N(3')-acetyltransferase III, aac(3)-II | -0.250909 |  | aminoglycoside |  |
| streptomycin phosphotransferase StrA | 0.1430005 |  | aminoglycoside |  |
| hypothetical protein aphA | -0.250838 |  | aminoglycoside |  |
| Beta-lactamase bla | 0.7418574 |  | beta-lactam | Jones‐Dias et al., 2016; |
| Beta-lactamase blaT TEM (plasmid) | -0.099589 |  | beta-lactam |  |
| Bleomycin resistance protein, ble(MBL) | 0.4537063 |  | beta-lactam, bleomycin | Dortet et al. 2012; |
| Penicillin-binding protein AmpH | 0.7229646 |  | beta-lactam, penicillin | Henderson et al., 1997; |
| Chloramphenicol O-acetyltransferase, cat | 0.105306 |  | chloramphenicol | Biswas et al.2012; |
| Chloramphenicol O-acetyltransferase, cat | 0.2201722 |  | chloramphenicol |  |
| Transposon Tn10 tetC protein | -0.09214 |  | tettracycline | Huang et al., 2015; |
| Tetracycline resistance protein, class E, tetB | 1.001805 | up | Tetracycline | Rather et al., 2012; |
| Fosmidomycin resistance protein, fsr | -0.27417 |  | fosmidomycin | Fujisaki et al. 1996 |
| Bifunctional polymyxin resistance protein ArnA | 0.0576627 |  | polymycin | Gatzeva-Topalova et al.,2004 |
| Acriflavine resistance protein A, acrF | -0.411972 |  | multiple antibiotic(ceftazidime) | Tânia Curiao et al., 2015; Tavı´o et al., 2014; |
| Antibiotic efflux pump membrane transporter ArpB | 1.0650665 | up | efflux | Vasiljevic et al., 1993; |
| Antibiotic efflux pump membrane transporter ArpB | 0.8410443 |  | efflux |  |
| Antibiotic efflux pump membrane transporter ArpB | -0.209172 |  | efflux |  |
|  |  |  | efflux |  |
| Multiple antibiotic resistance protein marR | -0.908557 |  | efflux | Tavı´o et al., 2014; |
|  |  |  | efflux |  |
| Macrolide export ATP-binding/permease protein MacB | -0.165807 |  | efflux | Kobayashi et al. 2001; |
| Macrolide export ATP-binding/permease protein MacB | -0.606656 |  | efflux | Kobayashi et al. 2001; |
| Macrolide-specific efflux protein macA | 0.0788778 |  | efflux | Kobayashi et al. 2001; |
| Drug resistance transporter, Bcr/CflA subfamily | -0.025771 |  | efflux | Smith et al. 2010 |
| Drug resistance transporter, EmrB/QacA subfamily | -2.376369 |  | efflux |  |
| Multiple antibiotic resistance protein marA | -1.162872 | down | efflux | Curiao et al., 2015 |
| Multiple antibiotic resistance protein marB | -1.23066 | down | efflux |  |
| Multidrug resistance protein B, emrB | -0.15632 |  | efflux | Lomovskaya et al.,1992; |
| Multidrug resistance protein D, emrD | -0.133186 |  | efflux | Nishino et al., 2001; |
| Multidrug resistance protein A, emrA | -0.368121 |  | efflux | Lomovskaya et al.,1992; |
| Multidrug resistance protein K, emrK | -1.028445 |  | efflux | Tanabe et al.,1997; |
| Multidrug resistance protein mdtA | -1.066098 | down | efflux | Nagakubo et al., 2002; |
| Multidrug resistance protein mdtB | 0.0040424 |  | efflux |  |
| Multidrug resistance protein mdtC | 0.727629 |  | efflux |  |
| Multidrug resistance protein mdtE | 2.7684241 | up | efflux | Nishino et al., 2008; |
| Multidrug resistance protein mdtG | 1.0784698 | up | efflux | Fàbrega et al., 2010; |
| Multidrug resistance protein mdtH | 0.3475574 |  | efflux | Xu et al., 2014; |
| Multidrug resistance protein mdtK | -0.625434 |  | efflux | Sato et al., 2013; |
| Multidrug resistance protein mdtL | -0.061093 |  | efflux | Hayes et al., 2006 |
| Multidrug resistance protein mdtM | -0.045395 |  | efflux | Holdsworth et al., 2013 |
| Multidrug resistance protein mdtN | -0.8358 |  | efflux | predictive |
| Multidrug resistance protein mdtO | 0.1414796 |  | efflux | predictive |
| Multidrug resistance outer membrane protein mdtP | 0.5565171 |  | efflux | Kim et al., 2009; |
| Multidrug resistance outer membrane protein mdtQ | 0.2256002 |  | efflux | Kim et al., 2009; |
| Cation efflux system protein CusC | -1.227225 |  | efflux | Franke et al., 2003; |
| Cation efflux system protein CusF | 0.5563474 |  | efflux |  |
| Cation efflux system protein CusB | 0.2579547 |  | efflux |  |
| Cation efflux system protein CusA | 0.3500476 |  | efflux |  |
| Antibiotic efflux pump membrane transporter ArpB, AcrB | -0.209172 |  | efflux | Aires et al., 2003; |
| Acriflavine resistance protein A, AcrA | -0.411972 |  | efflux |  |
| HTH-type transcriptional regulator AcrR | 0.3535622 |  | efflux |  |
| Outer membrane protein tolC | 0.4766412 |  | efflux | Lin et al., 2014; |
| Outer membrane protein C, OmpC | 1.0228155 | up | influx | Hancock et al., 1991; |
| Outer membrane protein F (Porin ompF) | -0.636492 |  | influx |  |
| Outer membrane pore protein E, phoE | -3.278744 |  | influx |  |

**Reference**

[Aires, J.R](https://www.ncbi.nlm.nih.gov/pubmed/?term=Aires%20JR%5BAuthor%5D&cauthor=true&cauthor_uid=15743938)., and [Nikaido, H](https://www.ncbi.nlm.nih.gov/pubmed/?term=Nikaido%20H%5BAuthor%5D&cauthor=true&cauthor_uid=15743938). (2005). Aminoglycosides are captured from both periplasm and cytoplasm by the AcrD multidrug efflux transporter of Escherichia coli. [*J Bacteriol.*](https://www.ncbi.nlm.nih.gov/pubmed/?term=AcrA+AND+gentamycin+transport) 187,1923-1929.

Biswas, T., Houghton, J.L., Garneau-Tsodikova, S., and Tsodikov, O.V. (2012). The structural basis for substrate versatility of chloramphenicol acetyltransferase CATI. *Protein Science.* 21, 520-530. doi: 10.1002/pro.2036

Curiao, T., Marchi, E., Viti, C., Oggioni, M.R., Baquero, F., and Martinez, J.L. (2015). Polymorphic Variation in Susceptibility and Metabolism of Triclosan- Resistant Mutants of Escherichia coli and Klebsiella pneumoniae Clinical Strains Obtained after Exposure to Biocides and Antibiotics. *Antimicrob Agents Chemother.* 59, 3413-3423. doi: 10.1128/AAC.00187-15

Fàbrega, A., Martin, R.G., Rosner, J.L., Tavio, M.M., and Vila, J. (2010). Constitutive SoxS expression in a fluoroquinolone-resistant strain with a truncated SoxR protein and identification of a new member of the marA-soxS-rob regulon, mdtG. *Antimicrobial agents and chemotherapy.* 54, 1218-1225. doi: 10.1128/AAC.00944-09

[Franke, S](https://www.ncbi.nlm.nih.gov/pubmed/?term=Franke%20S%5BAuthor%5D&cauthor=true&cauthor_uid=12813074)., [Grass, G](https://www.ncbi.nlm.nih.gov/pubmed/?term=Grass%20G%5BAuthor%5D&cauthor=true&cauthor_uid=12813074)., [Rensing, C](https://www.ncbi.nlm.nih.gov/pubmed/?term=Rensing%20C%5BAuthor%5D&cauthor=true&cauthor_uid=12813074)., and [Nies, D.H](https://www.ncbi.nlm.nih.gov/pubmed/?term=Nies%20DH%5BAuthor%5D&cauthor=true&cauthor_uid=12813074). (2003). Molecular analysis of the copper-transporting efflux system CusCFBA of Escherichia coli. [*J Bacteriol.*](https://www.ncbi.nlm.nih.gov/pubmed/12813074) 185, 3804-3812.

Fujisaki, S., Ohnuma, S., Horiuchi, T., Takahashi, I., Tsukui, S., and Nishimura, Y. (1996) Cloning of a gene from Escherichia coli that confers resistance to fosmidomycin as a consequence of amplification. *Gene.* 175, 83-87.

Gatzeva-Topalova, P.Z., May, A.P., and Sousa, M.C. (2004). Crystal structure of Escherichia coli ArnA (PmrI) decarboxylase domain. A key enzyme for lipid A modification with 4-amino-4-deoxy-L-arabinose and polymyxin resistance. *Biochemistry.* 43, 13370-13379.

Glenn, L.M., Englen, M.D., Lindsey, R.L., Frank, J.F., Turpin, J.E., and Berrang, M.E. (2012). Analysis of Antimicrobial Resistance Genes Detected in Multiple-Drug-Resistant Escherichia coli Isolates from Broiler Chicken Carcasses. *Microb Drug Resist.* 18, 453-463. doi: 10.1089/mdr.2011.0224

[Hancock, R.E](https://www.ncbi.nlm.nih.gov/pubmed/?term=Hancock%20RE%5BAuthor%5D&cauthor=true&cauthor_uid=1656859)., [Farmer, S.W](https://www.ncbi.nlm.nih.gov/pubmed/?term=Farmer%20SW%5BAuthor%5D&cauthor=true&cauthor_uid=1656859)., [Li, Z.S](https://www.ncbi.nlm.nih.gov/pubmed/?term=Li%20ZS%5BAuthor%5D&cauthor=true&cauthor_uid=1656859)., and [Poole, K](https://www.ncbi.nlm.nih.gov/pubmed/?term=Poole%20K%5BAuthor%5D&cauthor=true&cauthor_uid=1656859). (1991). Interaction of aminoglycosides with the outer membranes and purified lipopolysaccharide and OmpF porin of Escherichia coli. [*Antimicrob Agents Chemother.*](https://www.ncbi.nlm.nih.gov/pubmed/1656859) 35, 1309-1314.

Hayes, E.T., Wilks, J.C., Sanfilippo, P., Yohannes, E., Tate, D.P., and Jones, B.D. (2006). Oxygen limitation modulates pH regulation of catabolism and hydrogenases, multidrug transporters, and envelope composition in Escherichia coli K-12. *BMC microbiology.* 6, 89.

Henderson, T.A., Young, K.D., Denome, S.A., and Elf, P.K. (1997). AmpC and AmpH, Proteins Related to the Class C b-Lactamases, Bind Penicillin and Contribute to the Normal Morphology of Escherichia coli. *J Bacteriol.* 179, 6112-6121.

Holdsworth, S.R., and Law, C.J. (2013). The major facilitator superfamily transporter MdtM contributes to the intrinsic resistance of Escherichia coli to quaternary ammonium compounds. *J Antimicrob Chemother*. 68, 831–839. doi: 10.1093/jac/dks491

Huang, M., Qi, F., Wang, J., Xu, Q., and Lin, L. (2015). Changes of bacterial diversity and tetracycline resistance in sludge from AAO systems upon exposure to tetracycline pressure. *Journal of hazardous materials.* 298, 303-309. doi: 10.1016/j.jhazmat.2015.05.039

Jones-Dias, D., Manageiro, V., and Caniça, M. (2015). Influence of agricultural practice on mobile bla genes: IncI1‐bearing CTX‐M, SHV, CMY and TEM in Escherichia coli from intensive farming soils.*Environmental microbiology.* In press. doi: 10.1111/1462-2920.13021

Kim, J.Y., Inaoka, T., Hirooka, K., Matsuoka, H., Murata, M., and Ohki, R. (2009). Identification and characterization of a novel multidrug resistance operon, mdtRP (yusOP), of Bacillus subtilis. *Journal of bacteriology*. 191, 3273-3281. doi: 10.1128/JB.00151-09

Kobayashi, N., Nishino, K., and Yamaguchi, A. (2001). Novel Macrolide-Specific ABC-Type Efflux Transporter in Escherichia coli. *Journal of bacteriology.* 183, 5639-5644.

[Lin, C.W](https://www.ncbi.nlm.nih.gov/pubmed/?term=Lin%20CW%5BAuthor%5D&cauthor=true&cauthor_uid=24395237)., [Huang, Y.W](https://www.ncbi.nlm.nih.gov/pubmed/?term=Huang%20YW%5BAuthor%5D&cauthor=true&cauthor_uid=24395237)., [Hu, R.M](https://www.ncbi.nlm.nih.gov/pubmed/?term=Hu%20RM%5BAuthor%5D&cauthor=true&cauthor_uid=24395237)., and [Yang, T.C](https://www.ncbi.nlm.nih.gov/pubmed/?term=Yang%20TC%5BAuthor%5D&cauthor=true&cauthor_uid=24395237). (2014). SmeOP-TolCSm efflux pump contributes to the multidrug resistance of Stenotrophomonas maltophilia. [*Antimicrob Agents Chemother.*](https://www.ncbi.nlm.nih.gov/pubmed/?term=TolC+AND+gentamycin) 58, 2405-2408. doi: 10.1128/AAC.01974-13.

Lomovskaya, O., and Lewis, K. (1992). Emr, an Escherichia coli locus for multidrug resistance. *Proceedings of the National Academy of Sciences.*89, 8938-8942.

Nagakubo, S., Nishino, K., Hirata, T., and Yamaguchi, A. (2002). The Putative Response Regulator BaeR Stimulates Multidrug Resistance of Escherichia coli via a Novel Multidrug Exporter System, MdtABC. *JOURNAL OF BACTERIOLOGY.* 184, 4161–4167.

Nishino, K., and Yamaguchi, A. (2001). Analysis of a complete library of putative drug transporter genes in Escherichia coli. *Journal of bacteriology.* 183, 5803-5812.

Nishino, K., Senda, Y., and Yamaguchi, A. (2008). The AraC-family regulator GadX enhances multidrug resistance in Escherichia coli by activating expression of mdtEF multidrug efflux genes.  *J Infect Chemother.* 14, 23-29. doi: 10.1007/s10156-007-0575-y

Rather, M.A., Aulakh, R.S., Gill, J.P., Mir, A.Q., and Hassan, M.N. (2012). Detection and sequencing of plasmid encoded tetracycline resistance determinants (tetA and tetB) from food–borne Bacillus cereus isolates. *Asian Pacific journal of tropical medicine.* 5, 709-712. doi: 10.1016/S1995-7645(12)60111-4

Sato, T., Yokota, S., Uchida, I., Okubo, T., Usui, M., and Kusumoto, M. (2013). Fluoroquinolone resistance mechanisms in an Escherichia coli isolate, HUE1, without quinolone resistance-determining region mutations. *Frontiers in microbiology.*  4, 125.  doi: 10.3389/fmicb.2013.00125

Smith, K.P., Kumar, S., and Varela, M.F. (2009). Identification, cloning, and functional characterization of EmrD-3, a putative multidrug efflux pump of the major facilitator superfamily from Vibrio cholerae O395. *Archives of microbiology.* 191, 903-911. doi: 10.1007/s00203-009-0521-8

Tanabe, H., Yamasak, K., Furue, M., Yamamoto, K., Katoh, A., and Yamamoto, M. (1997). Growth phase-dependent transcription of emrKY, a homolog of multidrug efflux emrAB, genes of Escherichia coli, is induced by tetracycline. *The Journal of general and applied microbiology.* 43, 257-263.

Tavío, M.M., Aquili, V.D.,  and Vila, J, Poveda, J.B. (2014). Resistance to ceftazidime in Escherichia coli associated with AcrR, MarR and PBP3 mutations and overexpression of sdiA. *Journal of Medical Microbiology.* 63, 56–65. doi: 10.1099/jmm.0.063727-0

Vasiljevic, B., Fira, D., and Topisirovic, L. (1993). A genetic study of apramycin‐resistant mutants of Escherichia coli. *Journal of basic microbiology.* 33, 47-51.

Xu, C., Kong, X., Wang, H., Zhang, N., Kong, X., and Ding, X. (2014). MTDH mediates estrogen-independent growth and tamoxifen resistance by down-regulating PTEN in MCF-7 breast cancer cells. *Cellular Physiology and Biochemistry.* 33, 1557-1567. doi: 10.1159/000358719
