# Supplementary material for: Polyphosphate Kinase Mediates Antibiotic Tolerance in Extraintestinal Pathogenic Escherichia coli PCN033
Source: Front Microbiol. 2016 May 19;7:724. doi: 10.3389/fmicb.2016.00724 (PMC4871857; doi:10.3389/fmicb.2016.00724)
Supplement: Table S3 — Expression of genes implicated in biofilm. [file Table3.DOCX]

**Table S3.** Expression of genes implicated in biofilm.

| genes implicated in biofilm | total | up | down | Reference |
| --- | --- | --- | --- | --- |
| **appendages** |  |  |  |  |
| flagella cluster | 76 | 0 | 35 | Zhao et al., 2007 and Prigent-Combaret et al., 1999 |
| fimbriae cluster | 46 | 0 | 5 | Laverty et al., 2014 |
| curli cluster | 10 | 0 | 4 | Laverty et al., 2014 |
| biofilm PGA locus | 0 | 0 | 0 | Agladze et al., 2005 |
| waaWVL, LPS | 6 | 0 | 0 | Chassaing et al., 2015 |
| **positive regulators** |  |  |  |  |
| c-di-GMP diguanylate cyclase | 9 | 2 | 2 | Wu et al., 2015 |
| toxin-antitoxin system | 8 | 1 | 1 | Wood et al., 2014 |
| bolA, regulator of murein genes | 1 | 1 | 0 | Dressaire et al., 2014 |
| mcbR | 1 | 1 | 0 | Lord et al., 2014 |
| rssAB | 2 | 2 | 0 | Tsai et al., 2011 |
| **negative regulators** |  |  |  |  |
| c-di-GMP phosphodiesterase | 7 | 0 | 2 | Wu et al., 2015 |

**Reference**

Agladze， K., Wang， X., and [Romeo， T](https://www.ncbi.nlm.nih.gov/pubmed/?term=Romeo%20T%5BAuthor%5D&cauthor=true&cauthor_uid=16321928). (2005). Spatial periodicity of Escherichia coli K-12 biofilm microstructure initiates during a reversible, polar attachmentphase of development and requires the polysaccharide adhesin PGA. *J Bacteriol.*  187, 8237-8246.

[Chassaing, B](https://www.ncbi.nlm.nih.gov/pubmed/?term=Chassaing%20B%5BAuthor%5D&cauthor=true&cauthor_uid=25666140)., [Garénaux, E](https://www.ncbi.nlm.nih.gov/pubmed/?term=Gar%C3%A9naux%20E%5BAuthor%5D&cauthor=true&cauthor_uid=25666140)., [Carriere, J](https://www.ncbi.nlm.nih.gov/pubmed/?term=Carriere%20J%5BAuthor%5D&cauthor=true&cauthor_uid=25666140)., [Rolhion, N](https://www.ncbi.nlm.nih.gov/pubmed/?term=Rolhion%20N%5BAuthor%5D&cauthor=true&cauthor_uid=25666140)., [Guérardel, Y](https://www.ncbi.nlm.nih.gov/pubmed/?term=Gu%C3%A9rardel%20Y%5BAuthor%5D&cauthor=true&cauthor_uid=25666140)., and [Barnich, N](https://www.ncbi.nlm.nih.gov/pubmed/?term=Barnich%20N%5BAuthor%5D&cauthor=true&cauthor_uid=25666140). (2015). Analysis of the σE regulon in Crohn's disease-associated Escherichia coli revealed involvement of the waaWVLoperon in biofilm formation. [*J Bacteriol.*](https://www.ncbi.nlm.nih.gov/pubmed/?term=waaWVL+AND+Chassaing) 197,1451-1465. doi: 10.1128/JB.02499-14.

Dressaire, C., Moreira, R.N., Barahona, S., Alves de Matos, A.P., and Arraiano, C.M. (2015). BolA is a transcriptional switch that turns off motility and turns on biofilm development. *MBio.* 6, e02352-14. doi: 10.1128/mBio.02352-14.

Laverty, G., Gorman, S.P., and Gilmore, B.F. (2014). Biomolecular Mechanisms of Pseudomonas aeruginosa and Escherichia coli Biofilm Formation. *Pathogens.* 3,596-632. doi: 10.3390/pathogens3030596.

Lord, D.M., Uzgoren Baran, A., Soo, V.W., Wood, T.K., Peti, W., and Page, R. (2014). McbR/YncC: implications for the mechanism of ligand and DNA binding by a bacterial GntR transcriptional regulator involved in biofilm formation. *Biochemistry.* 53,7223-7231. doi: 10.1021/bi500871a

Prigent-Combaret, C., Vidal, O., Dorel, C., and Lejeune, P. (1999). Abiotic surface sensing and biofilm-dependent regulation of gene expression in Escherichia coli. *J Bacteriol.* 181,5993-6002.

Tsai, Y.H., Wei, J.R., Lin, C.S., Chen, P.H., Huang, S., and Lin, Y.C. (2011). RssAB signaling coordinates early development of surface multicellularity in Serratia marcescens. *PLoS One.*  6,e24154. doi: 10.1371/journal.pone.0024154

[Wood, T.L](https://www.ncbi.nlm.nih.gov/pubmed/?term=Wood%20TL%5BAuthor%5D&cauthor=true&cauthor_uid=26987441)., and [Wood, T.K](https://www.ncbi.nlm.nih.gov/pubmed/?term=Wood%20TK%5BAuthor%5D&cauthor=true&cauthor_uid=26987441). (2016). The HigB/HigA toxin/antitoxin system of Pseudomonas aeruginosa influences the virulence factors pyochelin, pyocyanin, and biofilm formation. [*Microbiologyopen.*](https://www.ncbi.nlm.nih.gov/pubmed/26987441)  doi: 10.1002/mbo3.346. [unpublished]

Wu, Y., Ding, Y., Cohen, Y., and Cao, B. (2015). Elevated level of the second messenger c-di-GMP in Comamonas testosteroni enhances biofilm formation andbiofilm-based biodegradation of 3-chloroaniline. *Appl Microbiol Biotechnol.*  99, 1967-1976. doi: 10.1007/s00253-014-6107-7

Zhao, K., Liu, M., and Burgess, R.R. (2007). Adaptation in bacterial flagellar and motility systems: from regulon members to 'foraging'-like behavior in E. coli. *Nucleic Acids Res.* 35,4441-4452.
