# Supplementary material for: Polyphosphate Kinase Mediates Antibiotic Tolerance in Extraintestinal Pathogenic Escherichia coli PCN033
Source: Front Microbiol. 2016 May 19;7:724. doi: 10.3389/fmicb.2016.00724 (PMC4871857; doi:10.3389/fmicb.2016.00724)
Supplement: Table S4 — Genes involved in biofilm formation (part results of RNA-seq). [file Table4.DOCX]

Table S4. Genes involved in biofilm formation (part results of RNA-seq)

| **genes involved in biofilm formation** | **log2(Fold_change) normalized** | **result** | **reference** |
| --- | --- | --- | --- |
| **flagella cluster** |  |  | Laverty et al., 2014, Zhao et al., 2007 and Prigent-Combaret et al., 1999 |
| Flagellar basal-body rod protein flgB | -9.633461867 | down |  |
| Flagellar basal-body rod protein flgC | -9.285636803 | down |  |
| Flagellar biosynthesis | -8.148683296 | down |  |
| RNA polymerase sigma factor for flagellar operon | -7.554887122 | down |  |
| Flagellar M-ring protein | -7.289364952 | down |  |
| Flagellar hook protein flgE | -7.042920792 | down |  |
| Flagellar hook-basal body complex protein fliE | -6.863706337 | down |  |
| Flagellar protein fliS | -6.680506105 | down |  |
| Flagellar hook-associated protein 2 | -6.551961789 | down |  |
| Flagellar protein fliT | -6.470974288 | down |  |
| Flagellar basal-body rod protein flgF | -6.444773815 | down |  |
| Flagellar basal-body rod protein flgG | -6.365323855 | down |  |
| Flagellar motor switch protein FliN | -6.32162965 | down |  |
| Flagellar hook-associated protein 1 | -6.290820794 | down |  |
| Flagellar protein export ATPase FliI | -6.191396149 | down |  |
| Flagellar fliJ protein | -6.083727854 | down |  |
| Flagellar protein FliL | -6.042326357 | down |  |
| Flagellar assembly protein fliH | -5.987239771 | down |  |
| Flagellar L-ring protein | -5.957703827 | down |  |
| Flagellar motor switch protein FliG | -5.746866612 | down |  |
| Flagellar biosynthetic protein FliQ | -5.681290392 | down |  |
| Flagellar motor switch protein FliM | -5.675424066 | down |  |
| Flagella basal body P-ring formation protein flgA | -5.644669904 | down |  |
| Flagellar biosynthetic protein flhB | -5.384797344 | down |  |
| Flagellar protein fliO | -5.276900137 | down |  |
| Flagellar P-ring protein | -5.194486986 | down |  |
| Flagellar brake protein YcgR | -4.889369022 | down |  |
| Flagellar hook-associated protein 3 | -4.811049729 | down |  |
| Flagellar biosynthetic protein fliP | -4.780658787 | down |  |
| Flagellar biosynthesis protein flhA | -4.482772504 | down |  |
| Flagellar hook-length control protein | -4.38183733 | down |  |
| Flagella synthesis protein flgN | -4.353005246 | down |  |
| Flagellar protein flhE | -3.39317701 | down |  |
| Flagellar biosynthetic protein fliR | -2.899930678 | down |  |
| Flagellar regulator flk | -1.021079589 | down |  |
| **c-di-GMP** |  |  |  |
| Cyclic di-GMP phosphodiesterase yahA | -1.806838771 | down | Wu et al., 2015 |
| Cyclic di-GMP phosphodiesterase YhjH | -6.392193689 | down |  |
| Diguanylate cyclase yddV | 1.238904204 | up |  |
| Oxygen sensor protein DosP | 1.543631956 | up |  |
| **regulators** |  |  |  |
| Protein BolA | 2.06394434 | up | Dressaire et al., 2014 |
| HTH-type transcriptional regulator mcbR | 1.845377475 | up | Lord et al., 2014 |
| NTE family protein rssA | 1.116970114 | up | Tsai et al., 2011 |
| Glucose-1-phosphate thymidylyltransferase RmlA | 1.143138044 | up | Zhuo et al., 2014 |
| protein Rnase III inhibitor YmdB | 1.057284455 | up | Kim et al., 2013 |
| **fimbriae** |  |  |  |
| Fimbrin-like protein fimI | -6.684423281 | down | Laverty et al., 2014 |
| Type-1 fimbrial protein, A chain, fimA | -7.967992695 | down |  |
| Chaperone protein fimC | -3.091301074 | down |  |
| Outer membrane usher protein fimD papC | -3.514241741 | down |  |
| Chaperone protein focC | -6.10959594 | down |  |
| **curli** |  |  |  |
| Curli assembly protein CsgC | -4.29523196 | down | Laverty et al., 2014 |
| Major curlin subunit | -1.166273545 | down |  |
| Minor curlin subunit | -1.884163362 | down |  |
| CsgBAC operon transcriptional regulatory protein | -3.135924065 | down |  |

**Reference**

Dressaire, C., Moreira, R.N., Barahona, S., Alves de Matos, A.P., and Arraiano, C.M. (2015). BolA is a transcriptional switch that turns off motility and turns on biofilm development. *MBio.* 6, e02352-14. doi: 10.1128/mBio.02352-14.

[Kim, T](https://www.ncbi.nlm.nih.gov/pubmed/?term=Kim%20T%5BAuthor%5D&cauthor=true&cauthor_uid=24267348)., [Lee, J](https://www.ncbi.nlm.nih.gov/pubmed/?term=Lee%20J%5BAuthor%5D&cauthor=true&cauthor_uid=24267348)., and [Kim, K.S](https://www.ncbi.nlm.nih.gov/pubmed/?term=Kim%20KS%5BAuthor%5D&cauthor=true&cauthor_uid=24267348). (2013). Escherichia coli YmdB regulates biofilm formation independently of its role as an RNase III modulator. [*BMC Microbiol.*](https://www.ncbi.nlm.nih.gov/pubmed/24267348) 13, 266. doi: 10.1186/1471-2180-13-266.

Laverty, G., Gorman, S.P., and Gilmore, B.F. (2014). Biomolecular Mechanisms of Pseudomonas aeruginosa and Escherichia coli Biofilm Formation. *Pathogens.* 3,596-632. doi: 10.3390/pathogens3030596.

Lord, D.M., Uzgoren Baran, A., Soo, V.W., Wood, T.K., Peti, W., and Page, R. (2014). McbR/YncC: implications for the mechanism of ligand and DNA binding by a bacterial GntR transcriptional regulator involved in biofilm formation. *Biochemistry.* 53,7223-7231. doi: 10.1021/bi500871a

Prigent-Combaret, C., Vidal, O., Dorel, C., and Lejeune, P. (1999). Abiotic surface sensing and biofilm-dependent regulation of gene expression in Escherichia coli. *J Bacteriol.* 181,5993-6002.

Tsai, Y.H., Wei, J.R., Lin, C.S., Chen, P.H., Huang, S., and Lin, Y.C. (2011). RssAB signaling coordinates early development of surface multicellularity in Serratia marcescens. *PLoS One.*  6,e24154. doi: 10.1371/journal.pone.0024154

Wu, Y., Ding, Y., Cohen, Y., and Cao, B. (2015). Elevated level of the second messenger c-di-GMP in Comamonas testosteroni enhances biofilm formation andbiofilm-based biodegradation of 3-chloroaniline. *Appl Microbiol Biotechnol.*  99, 1967-1976. doi: 10.1007/s00253-014-6107-7

Zhao, K., Liu, M., and Burgess, R.R. (2007). Adaptation in bacterial flagellar and motility systems: from regulon members to 'foraging'-like behavior in E. coli. *Nucleic Acids Res.* 35,4441-4452.

[Zhuo, C](https://www.ncbi.nlm.nih.gov/pubmed/?term=Zhuo%20C%5BAuthor%5D&cauthor=true&cauthor_uid=25285537)., [Zhao, Q.Y](https://www.ncbi.nlm.nih.gov/pubmed/?term=Zhao%20QY%5BAuthor%5D&cauthor=true&cauthor_uid=25285537)., and [Xiao, S.N](https://www.ncbi.nlm.nih.gov/pubmed/?term=Xiao%20SN%5BAuthor%5D&cauthor=true&cauthor_uid=25285537). (2014). The impact of spgM, rpfF, rmlA gene distribution on biofilm formation in Stenotrophomonas maltophilia. [*PLoS One.*](https://www.ncbi.nlm.nih.gov/pubmed/?term=RmlA+AND+Zhuo)  9, e108409. doi: 10.1371/journal.pone.0108409.
